# Cancer Cachexia: Underlying Mechanisms and Potential Therapeutic Interventions

**DOI:** 10.3390/metabo13091024

**Published:** 2023-09-20

**Authors:** Dean Directo, Sang-Rok Lee

**Affiliations:** Department of Kinesiology, New Mexico State University, Las Cruces, NM 88003, USA; ddirecto@nmsu.edu

**Keywords:** cachexia, proteolysis, inflammation, mitochondrial dysfunction, metabolic reprogramming, exercise

## Abstract

Cancer cachexia, a multifactorial metabolic syndrome developed during malignant tumor growth, is characterized by an accelerated loss of body weight accompanied by the depletion of skeletal muscle mass. This debilitating condition is associated with muscle degradation, impaired immune function, reduced functional capacity, compromised quality of life, and diminished survival in cancer patients. Despite the lack of the known capability of fully reversing or ameliorating this condition, ongoing research is shedding light on promising preclinical approaches that target the disrupted mechanisms in the pathophysiology of cancer cachexia. This comprehensive review delves into critical aspects of cancer cachexia, including its underlying pathophysiological mechanisms, preclinical models for studying the progression of cancer cachexia, methods for clinical assessment, relevant biomarkers, and potential therapeutic strategies. These discussions collectively aim to contribute to the evolving foundation for effective, multifaceted counteractive strategies against this challenging condition.

## 1. Introduction

Cancer cachexia, an irreversible, multifactorial syndrome, instigates alterations within various metabolic pathways across multiple organs and tissues [1,2]. It manifests in cancer patients, culminating in significant weight loss, accompanied by remarkable muscle wasting that is closely linked to tumor growth and inversely correlated with lifespan. This syndrome encompasses an array of symptoms, including anorexia, anemia, compromised immune function, and impaired physical function [3]. The repercussions of cancer cachexia are profound and far-reaching, impacting the clinical outcomes of cancer patients in numerous ways, such as impaired qualify of life, increased treatment failure risk, increased susceptibility to treatment side effects, and elevated mortality rates [4]. Approximately 20% of cancer-related deaths are attributed to cancer cachexia [1]. Although typically perceived as an end-of-life manifestation during the advanced stages of cancer, cachexia can also emerge at the initial phases of disease. Therefore, early identification and implementation of effective strategies to counteract cancer cachexia could yield myriad benefits, enabling patients to complete potentially curative chemotherapy, radiotherapy, immunotherapy, or surgery. A comprehensive evaluation of data from diverse institutions, comprising a retrospective analysis of 3047 cancer patients under clinical protocols by the Eastern Cooperative Oncology Group, unveiled a significant insight. Specifically, weight loss exceeding 5% of patient’s premorbid weight prior to commencing chemotherapy served as a predictive marker for early mortality, a prognostic indicator independent of disease stage and tumor histology [5]. Additionally, a discernible pattern of reduced response rates to chemotherapy emerged among patients who experienced weight loss. Hence, it is of paramount importance that investigations into the underlying mechanisms of cancer cachexia are channeled toward pioneering therapeutic strategies that adopt a multidisciplinary approach in search of an effective intervention for this challenging condition. This comprehensive review aims to delve into the existing body of literature elucidating the intricate mechanisms underpinning cancer cachexia while exploring potential therapeutic avenues for tackling this multifactorial syndrome.

## 2. Preclinical Models of Cancer Cachexia

This section delves into the synergistic utilization of rodent models in conjunction with emerging therapies to forge substantive advancements in the quest for efficacious treatments for cancer cachexia. By elucidating the intricate underpinnings of both cancer cachexia and cancer-related animal models, this section seeks to enhance comprehension. The arsenal of previous cancer cachexia animal models is characterized by diverse strategies, encompassing the introduction of cancer cells to the rodent through ectopic or orthotopic injections, utilization of human cancer cells or patient-derived xenograft, and creation of genetically modified mice prone to spontaneous tumor formation. These diverse models have laid the groundwork, propelling progress in unraveling the intricate mechanisms of cancer cachexia, with selected animal models standing out as primary contributors to this advancement [6]. The chosen animal models are often distinguished by their model-specific attributes that effectively isolate cachexia from other uncertain cancer-progressing phenomena. This deliberate isolation facilitates a focused exploration of the connections between alleviating cancer cachexia and extending survival time in the rodent models. As research continues to evolve, an array of emerging models is poised to enrich our understanding of cancer cachexia. Notable among these models are the APC^Min/+^ mouse, the colon-26 carcinoma mouse, the Lewis lung carcinoma mouse model, and various other genetically engineered counterparts. These models hold the potential to offer fresh insights into the complex landscape of cancer cachexia (Table 1).

### 2.1. APC^Min/+^ Mouse Model

The APC^Min/+^ mouse model, known in the research as a mouse model bearing multiple intestinal neoplasia that develops numerous adenomas is an established tool for studying intestinal and mammary tumorigenesis, has been extensively explored in the realm of colorectal cancer research [2,7,8,9,10,11]. This rodent model stands as a robust platform for comprehending both molecular intricacies and practical implications in cancer studies. Multiple intestinal neoplasia (Min) is a mutation of the murine adenomatous polyposis coli gene [12]. Min encodes a stop codon at codon 850 resulting in premature truncation of the polypeptide. This phenomenon is observed in germline mutations in certain cancer syndromes such as the Apc gene in humans with familial adenomatous polyposis (FAP) or Gardner syndrome (GS) [12]. The Apc^Min/+^ mouse model, a noteworthy exemplar, manifests cancer cachexia hinges on systemic interleukin-6 (IL-6) and has been meticulously employed to emulate the progression of colorectal tumor development akin to human familial adenomatous polyposis (a condition affecting the colon and rectum). A highlight of this model is the mutation of the Apc gene, a tumor-suppressor gene intricately linked to the Wnt signaling pathway that plays a pivotal role in regulating cellular proliferation, differentiation, and renewal [7,13].

Remarkably, Apc^Min/+^ mice follow a trajectory that mirrors their non-tumor-bearing counterparts (e.g., C57BL/6) until they reach approximately 12 weeks of age, at which point they begin to lose body weight [2]. By the time these mice reach 20 weeks of age, they experience a substantial loss of body mass, typically amounting to 20–25% compared to their peak weight or that of non-tumor-bearing counterparts. This model successfully replicates critical aspects of human cancer, including the progression of tumor burden, chronic inflammation, and the development of anemia [2]. Consequently, the Apc^Min/+^ mouse model closely replicates the course of cachexia observed in human cancer patients, rendering it a promising candidate as a preclinical model of cancer cachexia.

### 2.2. C26 Colon Carcinoma Model

The colon-26 (C26) colon carcinoma mouse model stands out as the most extensively examined animal model for cancer-induced cachexia [14,15,16,17,18,19]. The formation of tumors occurs as a result of subcutaneous injection or grafting of C26 carcinoma cells into the flank of BALB/c mice [14] or of CD2F1 mice [18]. Nevertheless, it is important to acknowledge a crucial limitation of this rodent model when investigating cancer cachexia—the manner in which C26 carcinoma cells are introduced extraneously might not faithfully replicate or simulate the intricate progression of cancer cachexia observed in humans [20,21]. Thus, this aspect warrants prudent consideration in the design of forthcoming studies on cancer cachexia. Additionally, the timeframe of inoculation in this model may not be optimally aligned with the desired experimental period for studying cancer cachexia in humans. Diverse conditions or distinct protocols can introduce variability, potentially compromising the reproducibility and robustness of research findings. These divergences are notably contingent on the timing of implementation. Previous research underscores that the divergent outcomes associated with the C26 rodent model are possibly linked to the strain of the mice—BALB/c or CD2F1 [14]—the sex of the mice [22], the specific implanted tumor type [14], the source of tumor, the dosage of C26 cells injected, and the precise site of injection [22].

### 2.3. Lewis Lung Carcinoma Model

The Lewis lung carcinoma (LLC) model represents a highly aggressive rodent carcinoma with a propensity for spontaneous metastasis in immunocompetent mice [23]. This model has emerged as a cornerstone for the exploration of metastatic progression [24,25], angiogenesis [25], and notably, cachexia [19]. A defining attribute of the LLC model is its innate capacity for cellular metastasis [19]. Additionally, this model demonstrates a swift and progressive decline in body and tissue mass, coupled with anorexia manifesting predominantly in the advanced phases of cancer progression [18]. A notable study by Kerr and colleagues delved into an intervention involving long-acting ghrelin, called EXT418, in the context of LLC-induced cachexia. The outcomes of their investigation revealed promising effects, including the mitigation of tumor-induced elevation of muscle IL-6 transcript levels and downregulation of Bcl-2/adenovirus E1B 19-kDa-interacting protein (BNIP3), a marker associated with mitophagy [26]. These findings underscored a reduction in skeletal muscle inflammation, proteolysis, and mitophagy, shedding light on potential avenues for intervention [26].

### 2.4. Other Genetically Engineered Models

In the realm of prospective genetically engineered models, Luan and colleagues developed a novel mouse model by utilizing transgenic female mice bearing ovarian tumors to enrich the understanding of cancer cachexia [27]. Their investigative focus encompassed a suite of biomarkers—activin A, growth differentiation factor 15 (GDF-15), IL-6, interleukin-1 beta (IL-1β), and tumor necrosis factor alpha (TNF-α)—which provided a comprehensive view of cancer cachexia’s unfolding. In an insightful revelation, the researchers established that their inventive mouse model effectively mimics the trajectory of human cancer-induced cachexia. Evident markers of this mimicry included muscle proteolysis, adipose tissue wasting, elevation of serum activin A and GDF-15, and the atrophy of both the pancreas and liver. Particularly noteworthy were the significant elevations in serum activin A and GDF-15—amounting to 76-fold and 10-fold increase, respectively—relative to baseline values as cancer-induced cachexia progressed [27]. Given that this mouse model recapitulated cardinal hallmarks of cancer cachexia, such as rapid loss of body weight, skeletal muscle atrophy, and adipose tissue depletion, these findings hold promise for steering future research into unraveling the intricate mechanisms underpinning cancer cachexia. Correspondingly, in the pursuit of a more faithful representation of human cancer cachexia, Talbert et al. engineered a mouse model of pancreatic ductal adenocarcinoma (PDA) termed KPP. This inventive model emulates the depletion of muscle and adipose tissues that parallels the advancement of tumor progression, presenting an opportunity to gain deeper insights into the intricate landscape of cancer cachexia [28].

**Table 1:** Preclinical Models of Cancer Cachexia.

Model Name	Notable Features	Reference
APC^Min/+^ Mouse Model	A highlight of this model is the mutation of the Apc gene, a tumor-suppressor gene intricately linked to the Wnt signaling pathway that plays a pivotal role in regulating cellular proliferation, differentiation, and renewal. This model successfully replicates critical aspects of human cancer, including the progression of tumor burden, chronic inflammation, and the development of anemia.	[7,13]
Colon-26 Carcinoma Model	The formation of tumors occurs because of the subcutaneous injection or grafting of C26 carcinoma cells into the flank of the rodent. The divergent outcomes associated with the C26 rodent model are possibly linked to the strain of the mice, the sex of the mice, the implanted tumor type, the source of the tumor, the dosage of the C26 cells injected, and the site of injection.	[14,18,22]
Lewis Lung Carcinoma Model	A defining attribute of this model is its innate capacity for cellular metastasis. This model demonstrates a swift and progressive decline in body and tissue mass, coupled with anorexia manifesting predominantly in the advanced phases of cancer progression.	[19]
Luan and Colleagues Mouse Model	A novel mouse model that utilizes transgenic female mice bearing ovarian tumors to study cancer cachexia. Notable biomarkers of this model are, growth differentiating factor 15, interleukin-6, interleukin-1 beta, and tumor necrosis factor alpha.	[27]
KPP Mouse Model	This inventive model emulates the depletion of muscle and adipose tissues that parallels the advancement of tumor progression of cancer cachexia.	[28]

## 3. Metabolic Reprogramming in Cancer Cachexia

Cancer cells may undergo metabolic reprogramming to meet their heightened energy demand and foster proliferation and metastasis. Simultaneously, they orchestrate significant molecular, cellular, and physical transformations within their host tissues to facilitate tumor advancement. Within the tumor microenvironment, a complex interplay of the following elements shapes tumor growth: (1) immune cells that play dual roles of either suppressing the tumor or promoting tumorigenesis; (2) stromal cells that secrete mediators that influence angiogenesis, proliferation, invasion, and metastasis; (3) blood vessel formation that supplies blood to the tumor cells; and (4) an extracellular matrix to support tumor growth, progression, and dissemination [29]. These components within the tumor microenvironment work in concert to promote tumor growth and concurrently contribute to cancer-induced muscle wasting. However, cancer cells must adapt to the microenvironment, particularly in response to hypoxic or hyponutrient conditions. Under hypoxic conditions, the tumor cell may continue to utilize glucose to generate adenosine triphosphate (ATP) through a phenomenon known as the Warburg Effect [30]. This phenomenon involves the production of ATP through aerobic glycolysis per unit of glucose, which is in contrast to the more efficient ATP production involving mitochondrial respiration [31,32]. Despite the lower ATP yield, the rate of glucose metabolism through aerobic glycolysis leads to rapid lactate production [33], which further contributes to the hypoxic microenvironment. The preference for aerobic glycolysis in cancer cells is attributed to its ability to support tumor growth even in hyponutrient environments [34]. Although less efficient in terms of ATP production, aerobic glycolysis generates ATP at a higher rate, which may be advantageous for sustaining tumor growth under nutrient-depleted conditions. Additionally, the hypoxic microenvironment induces changes that increases the demand for ATP-dependent membrane pumps, further promoting rapid aerobic glycolysis while maintaining relatively unchanged oxidative phosphorylation [35].

**Figure 1:**
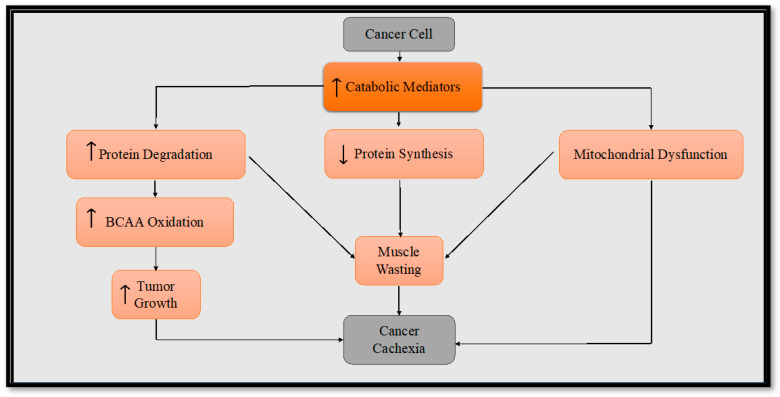
Diagram illustrating the underlying mechanisms of cancer cachexia. To sustain its growth, cancer calls release procachexic factors in the blood stream, thereby facilitating the progression of cancer cachexia. The procachexic factors trigger heightened protein degradation, reduced protein synthesis, and impaired mitochondrial function that all contribute to cancer cachexia. The escalated protein degradation in skeletal muscle boosts the availability of BCAAs. The tumor cells then harness this increased reservoir of BCAAs as a substrate to fuel their growth. The increased cellular growth of the tumor subsequently instigates the metastasis of the cancer cells, prompting their dissemination to various regions of the body.

In nutrient-scarce conditions, the tumor cells may resort to utilizing alternative substrates such as amino acids (e.g., glutamine, serine, arginine), fatty acids, and lipids to fuel their own proliferation [36]. Specifically, branched-chain amino acids (BCAAs: leucine, isoleucine, valine) are oxidized in skeletal muscle, accounting for as much as 20% during the course of cancer cachexia [37]. One avenue through which BCAAs contribute to tumor growth is their transportation into the tumor cell where they can directly activate mammalian target of rapamycin (mTOR) signaling for the tumor’s growth [38]. These amino acids can be converted to branched-chain α-keto acids via cytosolic branched-chain amino acid transaminase-1 (BCAT1) or mitochondrial BCAT2 in a reversible reaction. BCAT1 overexpression results in increased BCAA catabolism in cancer and is upregulated by several molecules (e.g., hypoxia-inducible factor 1, SMAD5, cMyc, Musashi2) although some cancers favor the reverse reaction [38,39]. Certain cancers reprogram the BCAA metabolism, altering the expression and activity of certain transporters and enzymes. This reprogramming favors the direct utilization of BCAAs while suppressing their catabolism, resulting in an accumulation of BCAAs within cancer cells [38]. This metabolic shift is likely due to changes in the expression and function of BCAA transporters and enzymes that participate in the BCAA metabolic pathway [39]. The buildup of BCAAs in these cancer cells is associated with the promotion of tumor growth via the activation of mTORC1 and the mTOR downstream signaling pathway [38,39,40]. The transformation of BCAAs into branched-chain a-keto acids generates glutamate as an additional energy source. Furthermore, these branched-chain a-keto acids can find utility within the mitochondria through their conversion into acetyl CoA and succinyl CoA, vital components that fuel the tricarboxylic acid (TCA) cycle, which ultimately contributes to the energy demands necessary for tumor growth [38]. Considering the aforementioned hypoxic and nutrient-deprived conditions present in the tumor microenvironment, it becomes evident that this metabolic reprogramming plays a crucial role in propelling the tumor cells into a hypermetabolic state. This hypermetabolic state not only supports tumor growth but also contributes to the complex interactions that foster both muscle wasting and tumor progression (Figure 1). By unraveling these intricate metabolic adaptations, we gain insights into potential therapeutic strategies that target the unique vulnerabilities of cancer cells and the associated complications they bring about.

## 4. Inflammatory Mediators in Cancer Cachexia

Under healthy physiological conditions, the immune systems serve as a defense mechanism, identifying potential threats such as pathogens or harmful agents and triggering an immune response. This response involves the release of proinflammatory cytokines that act upon the affected tissues, contributing to their repair and protection. In a similar fashion, tumor cells can release cytokines that support various aspects of tumor growth, such as those encompassing initiation, proliferation, angiogenesis, immunosuppression, metastasis, resistance to anticancer drugs, and energy supply within the tumor microenvironment [41]. Cytokines operate within the tumor microenvironment and can also interact with other body tissues, leading to systemic effects [42]. A considerable amount of evidence supports the role of cytokines in driving cellular processes that facilitate the initiation, promotion, invasion, and metastasis of cancer cells and consequently, the development of cancer-induced cachexia [43,44]. The production rate of several cytokines is closely linked to the occurrence of cachexia across multiple types of cancer [44].

Certain inflammatory mediators, such as C-reactive protein (CRP), IL-6, IL-1β, and TNF-α, play a pivotal role in initiating cachexia-induced muscle wasting [1,45,46,47,48]. Elevated systemic levels of IL-6, IL-1, and TNF-α in cancer patients seem to correlate with the progression of tumors [49,50,51,52]. These cytokines contribute to increased skeletal muscle protein degradation through the activation of three pathways: the ubiquitin-proteasome system (UPS) [53], the autophagy/lysosomal pathway [54], and the Ca^2+^-activated degradation pathway [55].

IL-6 is a widely recognized cytokine with significant implications for various biological functions. It plays a multifaceted role in tumor growth and metastasis by acting as a bridge between chronic inflammation and tumorigenesis and by contributing to muscle atrophy [56,57,58] and protein breakdown [59,60]. In the context of gastric cancer, a prognostic model identified as an RNA-binding protein called RNA-binding motif, single-stranded-interacting protein (RBMS1), was found to potentially serve as a potential promoter gene for metastasis in one study [61]. This study identified IL-6 as a crucial cytokine in RBMS1 overexpression, demonstrating its involvement in promoting migration and invasion of cancer cells through IL-6 transactivation and JAK2/STAT3 downstream signaling pathway activation. These findings shed light on the intricate molecular mechanisms underlying gastric cancer metastasis and its connection to IL-6 signaling. Further research in the field of colorectal cancer has unveiled insights into the influence of IL-6 on cancer cells. Specifically, in the microenvironment of early colorectal cancer tumors, cancer stem cells released by myofibroblasts contribute to the expansion of these cells. This process is mediated by IL-6 and IL-8, which induce hairy and enhancer of split (HES1) activation (a Notch signaling target) and activate the STAT3 pathway. These results suggest a significant interaction between cancer stem cells and the tumor microenvironment, offering potential avenues for targeted prevention and treatment strategies for colorectal cancers [62].

IL-1β is a notable biomarker related to muscle wasting in cancer cachexia [48]. This inflammatory mediator is closely linked to clinical manifestations of cachexia conditions in advanced cancer patients, such as weakness, loss of appetite, weight loss, and sarcopenia [48]. Elevated levels of IL-1β are particularly evident in patients with cancer-related anorexia, with its severity being closely correlated to the levels of IL-1β [63]. Moreover, the presence of increased IL-1β is connected to the loss of muscle mass, exacerbating anorexia and escalating energy expenditure [64]. In animal research, IL-1β has demonstrated a propensity to foster tumor growth through its promotion of angiogenesis, potentially hastening the progression of muscular weakness and weight loss. For instance, Voronov et al. demonstrated that elevated levels of IL-1β induce cancer cells to initiate and complete the processes of angiogenesis in mice with high levels of IL-1β and IL-1α inoculated with B16 melanoma cells and DA/3 mammary adenocarcinoma [65]. Similarly, Jung et al. demonstrated that IL-1β triggers upregulation of hypoxia-inducible factor-1α protein via a nuclear factor kappa B/cyclooxygenase-2 pathway, which subsequently enhances vascular endothelial growth factor (VEGF), a potent angiogenic factor required for tumor growth and metastasis [66]. These findings collectively underline the significance of IL-1β as a potential critical biomarker for diagnosing increased proteolysis and lipolysis in cancer cachexia. This notion is reinforced by additional research demonstrating the role of IL-1β and its strong association with cancer cachexia [48,67,68,69].

Tumor necrosis factor-alpha (TNF-*α*) is recognized for its multifaceted role as a growth factor, stimulating cellular growth and differentiation for various normal cells [70]. This molecule exhibits a broad range of biological activities, and clinical application has focused on inhibiting its effects to manage autoimmunity [71]. In instances of damage or infection, acute inflammation triggers a cascade of cytokines and chemokines, initiating an immune response to combat disrupted and harmed tissue. In the context of cancer cachexia, TNF-*α* serves as a proinflammatory cytokine and mediator of tumor-induced adipose and skeletal muscle wasting [72,73,74].

The role of TNF-α in cancer cachexia is twofold: directly inducing catabolism in skeletal muscle through the activation of the Nuclear Factor Kappa B (NF-kB) pathway and promoting ubiquitin-mediated proteasome degradation of muscle protein [47,75,76]. Beyond this, TNF-α contributes to cancer cachexia by fostering increased gluconeogenesis, adipose tissue loss, proteolysis, and reductions in protein and fat metabolism [75,76]. The catabolic effect of TNF-α was evidenced in a murine model, where tumor-bearing mice displayed substantially higher levels of TNF-α, atrogin-1, and muscle ring finger protein 1 (MuRF1) compared to healthy cohorts [77]. The increased atrogin-1 expression and activation of p38 MAPK pathway instigate muscle protein degradation and muscle atrophy [78]. Distinctions within the tumor microenvironment hold the potential to influence the progression of cancer cachexia by altering the array of circulating catabolic factors derived from the tumor milieu [77].

There is growing evidence that underscores the pivotal role of CRP in inflammatory processes and hosts’ response to pathways associated with infection. These pathways encompass apoptosis, phagocytosis, nitric oxide (NO) release, and the production of specific cytokines, such as IL-6 and TNF-α [79]. CRP stands as one of the most extensively studied inflammatory biomarkers linked to cancer cachexia, even serving as a proposed diagnostic criterion [80]. Its elevation is closely associated with weight loss, diminished quality of life, and shortened survival time among advanced cancer patients [81].

CRP is subject to regulation by key molecular triggers of cachexia, such as IL-6, IL-1β, and TNF-α, which not only promote cancer cell growth and safeguard cancer cells against apoptosis but also stimulate angiogenesis and metastasis [82,83]. Within this context, these proinflammatory cytokines stimulate the liver to synthesize CRP, further contributing to muscle wasting in cancer cachexia [82,84]. Elevated CRP levels are also correlated with an increased risk of cancer types such as liver, lung, colorectal, and breast cancer [85]. Among different cancer types, the highest mCRP levels have been observed in patients with esophageal, rectal, colon, bladder, or pancreatic cancer [86]. Emerging research demonstrates that elevated CRP levels can serve as an early indicator of severe lean tissue loss [86,87]. This evidence positions CRP as a promising early biomarker for cachexia and as a means to monitor the progress of anticachexia therapeutic interventions [87]. However, the significant limitation in the application of CRP as a biomarker for assessing cancer cachexia is the variability in cutoff values employed in previous research studies and the lack of standardization of recognizable cutoff values of CRP [46,86,87,88].

The existing body of evidence supporting the correlation between IL-8 and cancer cachexia is currently limited in its scope. To our knowledge, there were only two studies that reported an association between IL-8 and cancer cachexia. In the first study, a comparison of serum samples from normal healthy donors and pancreatic cancer donors revealed a positive correlation between serum IL-8 levels and catabolic conditions of cancer cachexia status, weight loss, and sarcopenia [89]. Similarly, Fogelman et al. proposed a regression model to predict cachexia levels based on inflammatory markers in pancreatic cancer patients, and IL-8 was among the markers considered [90]. IL-8, functioning as a chemokine (CXCL8), may hold potential as an element in antitumor treatment strategies. A review study exploring the efficacy of CXCL8 blockade and immune checkpoint inhibition therapy suggested that this combined intervention could serve as a possible antitumor strategy [91]. Despite the current limitations in research, the emerging connections between IL-8 and cachexia may offer intriguing avenues for future investigation and therapeutic interventions.

GDF-15, a member of the transforming growth factor beta (TGF-β) superfamily, was previously named as macrophage inhibitory cytokine-1 [92]. Under normal physiological conditions, GDF-15 expression is minimal; however, its expression varies across different health or disease states. For instance, lower plasma levels of GDF-15 have been associated with healthy aging, while elevated plasma levels of GDF-15 have been linked to poor overall health [93,94]. The introduction of GDF-15 expression mirrors events often observed in cancer cachexia, such as mitochondrial dysfunction, cellular stress, inflammation, aging, and other pathological conditions triggered by stress response cytokines [95]. GDF-15 is also known to affect inflammatory and apoptotic pathways [96,97]. Previous research has demonstrated that high circulating levels of GDF-15 are correlated with chronic inflammatory conditions including renal, lung, and cardiovascular diseases, as well as cancer [98]. Moreover, exosomes released by tumor cells have been implicated in the process of muscle wasting and fat lipolysis induced by cachexia [99,100,101]. These tumor-derived exosomes convey messages that facilitate tumor metastasis by influencing healthy or abnormal cells [102]. This suggests that increased levels of GDF-15 may be linked to elevated tumor exosome production, leading to proteolysis and lipolysis in cancer patients.

In the context of colorectal cancer, Wang et al. conducted a review analysis and noted that higher GDF-15 expression was associated with a low survival rate in NOD/SCID mice, strongly implicating GDF-15 as a prometastatic gene in colorectal cancer [103]. In support of this contention, Brown et al. reported that patients with high preintervention prostate cancer GDF-15 serum levels showed an eight-fold higher death rate than do those with lower levels [104]. Animal models investigating the association of GDF-15 and cancer-induced cachexia further underscore its significance. Wu et al. demonstrated that inhibiting GDF-15 in tumor-bearing mice (TOV21G) reversed body weight loss, muscle mass loss, and fat mass loss while degrading muscle function and impairing physical performance [105]. Similarly, Zhang et al. reported increased GDF-15 levels in C26 tumor-bearing mice, suggesting the potential role that GDF-15 in tumor-derived exosomes have in facilitating muscle atrophy and pointing to the possibility of targeting GDF-15 for cancer cachexia treatment [99].

## 5. Skeletal Muscle Alteration in Cancer Cachexia

### 5.1. Protein Synthesis

Protein synthesis plays a crucial role in addressing the wasting of skeletal muscle during the progression of cancer. The process of protein synthesis is primarily regulated by the initiation phase of protein translation and has two important control points [106]. The first control point is the binding of initiator methionyl-tRNA or met-tRNA to the 40S ribosomal subunit that is regulated by the eukaryotic initiation factor 2 (elF2) [106]. The second control point of translation initiation is the recruitment of 40S ribosomal subunit to mRNA that is mediated by the eukaryotic initiation factor 4 (elF4) complex [106]. Muscles possess the ability to synthesize protein in response to certain stimuli, including energy status, anabolic hormones, catabolic hormones, and loading of the musculature [107]. A major player in regulating these growth-related stimuli and myofiber size is the PI3K/Akt/mTOR pathway, known for its influence on the interaction between insulin-like growth factor-1 (IGF-1) and the forementioned pathway [8]. However, protein synthesis in muscle is suppressed, contributing to the progression of muscle wasting under cancer-induced catabolic environments. For instance, as cancer cachexia advances, both circulating IGF-1 and muscle IGF-1 gene expression tend to decrease [108], thereby contributing to the decrement of protein synthesis. This contention has been studied in various human and animal models evidenced elsewhere in this review, yielding varying degrees of efficacy. The ongoing pursuit to manage cancer cachexia revolves around identifying interventions capable of addressing the alterations in protein synthesis that transpire in the advanced stages of cancer cachexia.

### 5.2. Muscle Proteolysis

The acceleration of skeletal muscle loss in cancer cachexia stems from the upregulation of catabolic factors, such as the ubiquitin-proteasome system, myostatin, and apoptosis-inducing factors [109], coupled with the downregulation of anabolic factors, including IGF-1 and its activation of the PI3K/Akt/mTOR signaling pathway [110]. This interplay between catabolic and anabolic signaling disrupts the delicate balance in skeletal muscle, ultimately leading to muscle wasting in cancer cachexia. Central to this process, the ubiquitin–proteasomal system (UPS) stands as the main degradative pathway mediating the progressive protein loss in cachexia [111]. The UPS involves a series of enzymes that modify specific protein substrates through ubiquitin tagging, which is followed by the proteolysis of these ubiquitin-labeled substrates by 26S proteasomes [112]. This ubiquitin conjugation to the substrate is facilitated by a multistep cascade reaction of the ubiquitin-activating enzymes (E1), ubiquitin-conjugating enzyme (E2), and a ubiquitin ligase (E3) [112]. In the context of muscle atrophy in cancer cachexia, two muscle-specific E3 ubiquitin ligases, atrogin-1/MAFbx and muscle ring finger protein 1 (MuRF-1), play pivotal roles in driving muscle proteolysis and wastage. The activation of forkhead box O (FOXO) family transcription factors further enhances the expression of MuRF-1 and atrogin-1, thus intensifying protein proteolysis and muscle degradation [1,75,113]. FOXO activation is mediated by cytokines such as TNF-α, IL-1, and proteolysis-inducing factor (PIF) [1,6,75]. These cytokines also contribute to the p38 and JAK/mitogen-activated protein kinase (MAPK) cascade that plays a key role in apoptosis [1].

Apoptosis, also known as “programmed cellular death”, is a vital process essential for both normal developmental processes and the homeostasis of multicellular organisms. A group of proapoptotic caspases, including caspase-2, -3, -6, -7, -8, -9, and -10, play a crucial role in transmitting signals for cellular death. In parallel, proinflammatory caspases, such as caspase-1, -4, -5, -11, and -12, are responsible for regulating cytokine maturation during inflammatory responses [114,115]. Activation of initiator caspases initiates a cascade of downstream caspase activities that ultimately lead to cellular death.

In cancer cachexia, there is a significant increase in the activity of caspase-1, -3, -8, and -9 in the cachectic skeletal muscle of tumor-bearing mice [116]. Upregulation of caspase-8 and -9 has been observed in cancer cachexia patients, promoting the activation of the final executioner of the apoptotic signaling pathway, caspase-3 [117]. This activation of caspase-3 subsequently triggers protein loss, thereby contributing to the progression of cancer cachexia [118]. Additionally, muscle atrophy and disease are associated with the loss of myonuclei, as indicated by Allen and colleagues, which further exacerbates the advancement of cancer cachexia [119]. Supporting this notion, D’Emilio et al., who conducted electron microscopy studies on skeletal muscle samples from cancer patients, demonstrated apoptosis-related morphological changes in myonuclei [120].

Maintaining skeletal muscle homeostasis relies on autophagy to clear damaged proteins and organelles. This process involves the formation of autophagosomes, which are double-layered vesicles that eventually fuse with lysosomes, where intracellular materials are broken down [121]. In the context of cancer cachexia, cytokine release from cancer and inflammatory cells can disrupt autophagy balance, mitophagy, and related signaling pathways that contribute to disease progression [122]. The modulation of autophagy genes can stimulate autophagy pathways, resulting in increased skeletal muscle breakdown [123]. One study reported that altered autophagy, whether excessive or defective, played a role in muscle atrophy in cancer cachexia across different cachexia models [124]. Elevated autophagy also affects the mitochondria, leading to reduced mitochondrial content, and ultimately resulting in reduced capacity in atrophied muscle [123]. This is attributed to the damaged mitochondria that are unable to execute oxidative phosphorylation efficiently [125]. Damaged mitochondria are isolated from healthy mitochondrial networks via fusion or fission and are then targeted for degradation through autophagy [125]. Furthermore, mitochondrial dysfunction can arise from disrupted coordination between mitochondrial fusion and fission as observed in cancer cachexia patients with altered indices of mitochondrial fission and fusion [126] as well as in tumor-bearing mice [127].

## 6. Altered Mitochondrial Metabolism in Cancer Cachexia

Five potential mechanisms may impair mitochondria function, thereby contributing to cancer cachexia. First, cancer is linked to DNA mutations that impact mitochondria, stemming from modifications to subunits within the electron transport chain [128]. This connection was demonstrated in tumor-bearing mice, revealing alterations in all four complexes of the electron transport system [129]. These alterations contribute to impaired mitochondria exhibiting diminished oxidative phosphorylation capabilities.

Second, the generation and progression of cancer toward malignancy are primarily triggered by oxidative stress induced by reactive oxygen species (ROS) [130]. ROS are produced by mitochondria that release superoxide as a byproduct of oxidative respiration [131]. Additionally, mitochondrial ROS (mROS) can be generated either in the citric acid cycle or in the electron transport chain [132]. Increased levels of ROS are often found in cancer cells due to increased metabolic activities and altered antioxidant capacities [133,134,135]. While ROS promotes tumorigenesis, elevated ROS can also be cytotoxic [136]. In particular, the hyperproliferation of tumor cells is associated with heightened ROS generation; however, these cells can adapt to flourish even when oxidative stress disrupts the redox balance, pushing it away from a reduced state. Tumor cells achieve this by bolstering their antioxidant defenses, thereby optimizing ROS-driven proliferation while simultaneously avoiding ROS thresholds that would induce senescence, apoptosis, or ferroptosis [137,138].

A third potential characteristic of dysfunctional mitochondria is increased glycolytic activity with impaired mitochondrial oxidative phosphorylation, characteristics seen similarly in cancer cachexia [100]. This has previously been termed “metabolic flexibility”, wherein individual cancer cells may exhibit variability in their metabolic phenotype [139,140]. The alterations in the metabolic phenotype within cancer cells grant them the capability to swiftly modify their energy production mechanisms from mitochondrial oxidative phosphorylation to accelerated glycolysis to support tumor cell growth, particularly when the tumor microenvironment shifts from normoxia to hypoxia [140]. However, it is worth noting that the overall ATP production in cancer cells is not exclusively ascribed to accelerated glycolysis (approximately up to 50–60%) but also coincides with mitochondrial oxidative phosphorylation [141]. Thus, a combination of glycolysis and mitochondrial oxidative phosphorylation contributes to overall ATP generation in cancer cells to sustain their growth [140]. This phenomenon was substantiated by Herst et al., who elucidated the transformation of energy production for tumor cell growth changes from oxidative phosphorylation to expedited glycolysis, which resulted in diminished overall ATP production within the hypoxic environment [142]. It may be inferred that excess ROS may contribute to this glycolysis dysregulation and vice versa [143]. Moderate and transient elevation of ROS levels can prompt glucose uptake [143]. However, if ROS concentrations surge excessively and/or persist over extended periods, a vicious circle of ROS-stimulated glucose uptake and glucose-stimulated ROS production can be triggered [143]. Within cancer cells, the increased ROS production arising from metabolic dysregulation, and swift proliferation may induce an amplification of an antioxidant capacity, enabling both heightened ROS production and effective elimination to retain the ROS levels below the threshold for cell demise. This orchestrated interplay aids in sustaining cancer cell survival [140].

Fourth, mitochondria are directly involved in the regulation of cellular death, including apoptosis and necrosis processes [144]. Mitochondria also control necroptosis, a regulated form of necrosis that needs ROS generation and depends on mitochondrial permeability transition [145]. Mitochondria may also undergo certain processes in order to maintain cellular homeostasis. One such process, called autophagy, is the catabolic action of recycling or removing dysfunctional or decaying cells. The process of maintaining mitochondrial homeostasis in which mitochondria are specifically targeted for degradation is called mitophagy [146]. Mitophagy occurs to alleviate oxidative stress and prevent carcinogenesis. However, in low oxygen conditions or low nutrient availability, mitophagy can protect cells from apoptosis and support tumor cell survival [146]. Thus, by inhibiting the mitophagy of mitochondria that leads to the resulting decrease in mitochondrial metabolism, tumor cell death may be promoted and further augmented by ROS [146]. Additionally, this pathway may provide insight into future therapies for promoting tumor cell death.

Fifth, metabolic reprogramming is also linked to mutations in several genes that encode enzymes of the citric acid cycle, which facilitate malignant transformation [147]. Regarding heightened glycolytic activity in cancer cachexia, specific oncogenes contribute to the regulation and modulation of its metabolism. For instance, oncogenes such as phosphatidylinositol 3-kinase (PI3K) [148,149,150] and hypoxic inducing factor (HIF) [151,152] foster increased glycolytic activity in cancer cachexia are also linked to tumor progression and resistance to cancer therapies [146]. Moreover, a distinctive trait of all tumors is continuous cellular proliferation driven by numerous molecular alterations [153], which in turn contributes to compromised mitochondrial function.

The process of mitochondrial biogenesis which occurs mainly in healthy cells is regulated by peroxisome proliferator-activated receptor gamma (PPARγ) coactivators [154]. Peroxisome proliferator-activated receptor gamma co-activator 1-alpha (PGC-1α), a master regulator of mitochondrial biogenesis and oxidative mechanism, is highly expressed in skeletal muscle during exercise, stimulating IGF-1 activation [155] while repressing myostatin signaling [156]. The PGC-1α isoform is expressed in metabolically active tissues, such as those of the liver, kidneys, and brain, and responds to energy-demanding situations (e.g., exercise). The overexpression of PGC-1α increases the amount of mitochondrial DNA, which encodes several protein subunits of the mitochondrial respiratory chain [154]. PGC-1α protects against ROS-induced cellular death through the upregulation of antioxidant enzymes [157].

Animal studies demonstrated that skeletal muscle-specific transgenic expression of PGC-1α4 exhibited increased muscle mass and strength and dramatic resistance to the muscle wasting in cancer cachexia [158]. A study revealed that aerobic exercise such as swimming and treadmill running raised PGC-1α content in the muscle by 75% after the discontinuation of aerobic exercise and 95% 6 h after swimming [159]. The increase of PGC-1α in response to exercise was also evidenced in humans. In addition, A 4-week regimen of knee extensor exercise led to an increase in PGC-1α transcription and mRNA content in the skeletal muscle of healthy individuals [160]. The utilization of PGC-1α knockout mice to explore its impact on organelle function revealed diminished mitochondrial content in both white and red muscles, alongside disrupted mitochondrial function and heightened susceptibility to apoptosis as evidenced by apoptotic signaling and cytochrome-c oxidase activity [161]. Further, PGC-1α upregulation with exercise appears to enhance muscle mass and quality while counteracting cancer-induced muscle wasting [44]. In a murine model of Apc^Min/+^ mice, it was discovered that the progression of cancer cachexia led to the suppression of PGC-1α expression in the gastrocnemius and soleus muscles at 20 weeks of age [60]. On the other hand, significant increases in both protein and gene expression levels of PGC-1α were observed in C26 mice and LLC tumor-bearing mice when treadmill running was combined with erythropoietin treatment [162]. The alterations in the PGC-1α expression levels could potentially impact the prognosis of cancer cachexia. Thus, the expression levels of PGC-1α might serve as a feasible biomarker for diagnosing the severity or stage of cachexia.

## 7. Myokines as Potential Therapeutic Agents for Cancer Cachexia

### 7.1. Myostatin

Myostatin is a highly conserved member of the transforming growth factor-beta family in skeletal muscle [163]. Myostatin has been identified as a negative regulator of myogenesis that inhibits myoblast proliferation, leading to a decrease in muscle growth [164]. Once myostatin binds to its type I receptors, Activin-4 (ALK-4) or Activin-5 (ALK-5), and type II receptors, Activin Receptor II A (ActRIIA) or Activin Receptor II B (ActRIIB), intracellular signaling is initiated via phosphorylation and activation of the transcription factors Smad2 and Smad3, the primary transcription factors in the myostatin pathway, leading to the loss of muscle mass [165,166,167]. Furthermore, myostatin-Smad2/3 signaling has been shown to inhibit the effect on the IGF1–Akt–mTOR signaling pathway, further alluding to the possibility that communication between myostatin and the IGF1–Akt–mTOR signaling pathway can control the degree of muscle fiber hypertrophy [168].

In regard to cancer cachexia, myostatin plays an integral role in muscle mass regulation, potentially contributing to accelerated muscle loss in this catabolic condition. Notably, previous research has demonstrated that mutations or absence of myostatin leads to significant muscle growth in humans and vertebrate animals [169,170,171,172,173]. Moreover, several studies have indicated that inhibition of myostatin could potentially help preserve skeletal muscle in tumor-bearing animal models [174,175,176,177] as well as in cancer patients [178]. A recent study demonstrated the protective effect of myostatin inhibition by attenuating soluble ActRIIB, which prevented not only skeletal muscle loss but also cancer-induced cardiac muscle atrophy [179]. Winbanks exhibited this phenomenon through the delivery of Smad7 gene therapy in mouse models of cachexia, as Smad7 functions as an intracellular negative regulator, curbing the activation of Smad2 and Smad3 while promoting the degradation of ActRIIB complexes [180]. Additionally, the administration of Smad7 was shown to effectively suppress Smad2/3 signaling downstream of ActRIIB and hinder the expression of atrophy-related ubiquitin ligases such as MuRF1 and F-box (MAFbx) [180]. Given these research findings, the implementation of myostatin inhibitor strategy aimed at reducing myostatin levels may hold promise for enhancing muscle mass in those with cancer-induced muscle wasting.

Previous research has shown that the implementation of a myostatin inhibitor in conjunction with aerobic exercise implemented via treadmill running in healthy mice exhibited improved physical function as seen in increased treadmill running time and distance to exhaustion, improved metabolic rates, and significantly improved insulin sensitivity [173]. Additionally, the inhibition of myostatin from this study was associated with a decrease of Smad3 phosphorylation and increased PGC-1α expression as well as decreased MuRF-1 [173]. When administered in mice inoculated with LLC cells, the treated mice show significantly improved muscular atrophy through the inhibition of the myostatin and Smad signaling pathway resulting in lowered muscle atrophy mediators such as MuRF1, F-box only protein 31, and MAFbx/atrogin-1 [176]. Further work has been documented in both human and rodent models in individuals with advanced stage cancer and healthy individuals [181]. Although investigating myostatin as a potential avenue for intervening in the preservation of muscle mass in cancer cachexia holds promise, its translation to human applications remains uncertain due to the lack of specificity and potential toxicities in clinical patients [182]. Despite these challenges, it is crucial that future formulations of anticancer therapies persist in examining myostatin as an encouraging molecular target for addressing muscle waste.

### 7.2. Fibroblast Growth Factor 21

Fibroblast Growth Factor 21 (FGF-21) is a signaling protein with diverse biological functions in development and metabolism. FGF-21 levels increase with hunger, stress, mitochondrial dysfunction, obesity, mitochondrial myopathies, and aging [183,184]. Holm and colleagues found that FGF-21 levels, independently associated with IL-6 and lower muscle mass, were significantly higher in patients with cardiac cachexia than in healthy cohorts [185], suggesting that the increased plasma levels of FGF-21 in patients with cardiac cachexia correlate with inflammation and muscle wasting [185]. Thus, it can be speculated that elevated systemic FGF-21 levels may contribute to the increased inflammation seen in cancer cachexia. In further support of FGF-21 as a potential contributor to cancer cachexia, Franz and colleagues reported that cachectic patients had significantly higher total FGF-21 levels than did their healthy control counterparts, indicating an association between cachexia and FGF-21 that was independent of sex, age, and body mass index [186]. Further, Oost et al. discovered that the ablation of the FGF-21 gene protected mice from muscle loss and weakness during fasting. These mice exhibited maintained protein synthesis rates and a reduction in the mitochondrial protein BNIP3 that plays a crucial role in mitochondrial autophagy [187]. They also highlighted the overexpression of FGF-21 prompted autophagy and led to a 15% reduction in muscle mass, which underscored the significance of BNIP3 inhibition in shielding against FGF21-dependent muscle wasting in adult animals [187].

### 7.3. Interleukin-15

Interleukin-15 (IL-15) is a myokine abundant in skeletal muscle and is known for its anabolic effect on muscle protein metabolism. IL-15 accumulation in skeletal muscle in response to exercise training solidifies its classification as a myokine [188,189,190]. A strategy to increase IL-15 levels has emerged as a potential anticachectic therapy, owing to its anabolic effect on muscle protein metabolism. Notably, previous research indicated that IL-15 fosters the proliferation of T, B, and natural killer cells, stimulating the expression of stem, central, and effector memory CD8 T cells [191]. These cells play a pivotal role in protecting the host from autoimmune diseases by suppressing self-reactive cells (T cells) [192]. They also positively influence immune response and inflammation through antibody production and promote T-cell activation and proliferation through antigen presentation (B cells) [193]. Furthermore, they direct their attention toward infected or cancerous cells [194]. Although IL-15 does not directly contribute to cancer cachexia pathogenesis, the increase in these cells may lead to an augmented immune response associated with IL-15 and potentially serve as a promising biomarker in the treatment of cancer cachexia.

In terms of cancer cachexia progression, research into the relationship between IL-15 and cancer cachexia is still in its early stages. Studies involving cancer patients undergoing anticancer therapies revealed that those patients who gained weight exhibited increased IL-15 values at 4 and 8 weeks during treatment, compared to both their baseline levels and patients who lost weight [195]. This rise in IL-15 values among patients who gained weight, along with its correlation with body weight and muscle mass, suggests a possible connection between IL-15 and body composition in cancer cachexia patients. In one study, IL-15 administration on male Wistar rats with intraperitoneal inoculation of 10^8^ AH-130 Yoshida ascites hepatoma cells led to a decrease in protein degradation rates compared to non-tumor-bearing mice [196]. Additionally, aerobic exercise and an antioxidant treatment (selenium nanoparticle supplementation) in 4T1 breast cancer cachectic mice resulted in increased food intake and preservation of muscle mass in the tumor-bearing mice. These results may be attributed to the modifications in the balance of anti-inflammatory mediators such as interleukin 10 (IL-10) and TNF-α ratios, along with IL-15 expression within skeletal muscle [197]. These studies collectively enhance our understanding of the mechanisms underlying IL-15’s preventive influence and its potential role as a biomarker for body composition in individuals with cancer cachexia. Furthermore, they shed light on its potential application in strategies aimed at mitigating accelerated muscle waste during cancer treatment.

## 8. Dysfunction of Adipose Tissue in Cancer Cachexia

The progression of cancer cachexia is driven by the simultaneous depletion of skeletal muscle and adipose tissue. Although the evidence of muscle wasting in cancer cachexia is well documented, the intricate mechanisms behind the loss of adipose tissue in this context remain limited. In certain cancers that progress toward cachexia, the release of various cytokines, such as TNF-α, IL-6, and interferon gamma (IFN-γ), plays a significant role [198]. Notably, adipose wasting often occurs prior to the loss of muscle mass in the early stages of cancer cachexia [198,199].

The heightened inflammatory response targeting adipose tissue can be attributed to the transformation of white adipose tissue into brown adipose tissue or the dysregulation of white adipose tissue. Studies in animal models demonstrated that chronic inflammation leads to increased expression of uncoupling protein 1 (UCP1), a protein that facilitates nonshivering thermogenesis in mammals and promotes the transition from white adipose tissue to brown adipose tissue [200,201]. As the conversion of white adipose tissue to brown adipose tissue progresses, the brown adipose tissue becomes a significant contributor to increased lipid mobilization and energy expenditure [200,202,203]. This heightened energy expenditure results from the increased thermogenesis of brown adipose tissue, which redirects energy from food toward heat generation rather than toward ATP synthesis. [204]. Supporting this, Petruzelli et al. found that increased UCP1 expression led to greater lipid mobilization and energy expenditure in cachectic mice [201]. Similarly, in a study involving human cancer patients, those with cancer cachexia exhibited a higher expression of UCP1 in their adipose tissue compared to cancer patients without cachexia [201]. Therefore, research focused on understanding abnormal lipid metabolism and preserving adipose tissue and its function in cancer cachexia patients could be a promising avenue to mitigate the loss of body weight as cancer cachexia progresses.

## 9. Potential Therapeutic Interventions for Cancer Cachexia

### 9.1. Clinical Care in Cancer Cachexia

**Figure 2:**
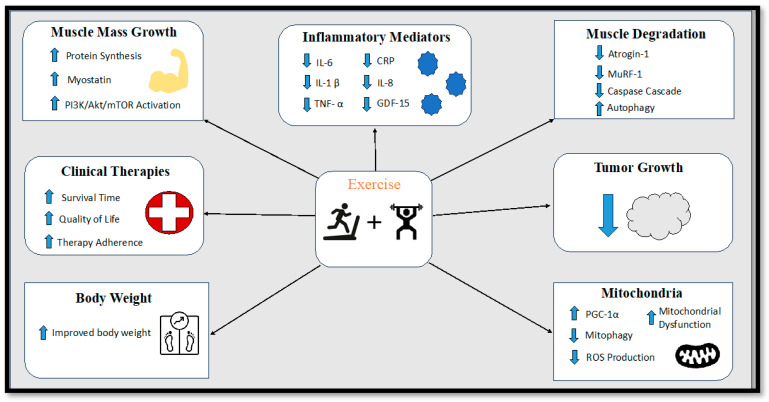
Diagram displaying the effects of exercise training on cancer cachexia. IL-6 = Interleukin-6, IL-1β = Interleukin-1 beta, TNF-α = Tumor Necrosis Factor Alpha, CRP = C-reactive protein, IL-8 = Interleukin-8, GDF-15 = Growth Differentiation Factor 15, PI3K/Akt/mTOR = phosphoinositide 3-kinase (PI3K)/protein kinase B (Akt)/ mammalian target of rapamycin (mTOR) pathway, MuRF-1 = Muscle ring-finger protein-1, PGC-1α = Peroxisome proliferator-activated receptor-gamma coactivator-1 alpha.

The overarching objective in the management of cancer cachexia is to achieve a cure or reversal of skeletal muscle loss and body weight decline. Nonetheless, the intricate nature of cancer-induced cachexia introduces formidable challenges to both its diagnosis and treatment. Consequently, the task of addressing cancer cachexia necessitates multifaceted approaches that can effectively target the loss of skeletal muscle and body weight, thereby enhancing the overall quality of life and survival prospects for cancer patients. The constellation of attributes associated with cancer cachexia encompasses a spectrum of issues, including insufficient food intake, weight reduction, depletion of muscle mass, diminished muscular strength, escalated catabolism, premature satiety, alterations in taste perception, nausea, bodily discomfort, diminished concentration, and chronic fatigue [75]. A comprehensive strategy for treating cancer cachexia should encompass a range of interventions, integrating pharmacological, nutritional, and exercise-based approaches to address the unmet medical requirements posed by this complex condition [205,206,207]. Initial efforts to counteract cancer cachexia focused on preventing body weight loss and were based on the premise that preserving body weight could impede the progression of cachexia. However, this approach proved challenging to implement in clinical settings due to the lack of standardized protocols for assessing, treating, and monitoring the progression of cancer cachexia. To the best of our knowledge, a universally accepted and singularly standardized treatment for cancer cachexia that effectively stabilizes or reverses its impacts has yet to be established. Despite this, the subsequent sections will delve into potential therapeutic avenues that warrant exploration and consideration as viable strategies in the battle against cancer cachexia (Figure 2).

### 9.2. Nutritional Interventions

#### 9.2.1. Omega-3 Polyunsaturated Fatty Acids

Omega-3 Polyunsaturated fatty acids (n-3), consisting of a mixture of two vital components, eicosapentaenoic acid (EPA) and docosahexaenoic acid (DHA), represent a class of anti-inflammatory supplements renowned for their anticatabolic properties. The Food and Nutrition Board of the Institute of Medicine (IOM) recommends a daily n-3 intake of 1.6 g for men and 1.1 g for women to ensure nutritional adequacy [208]. The Dietary Guidelines for Americans (DGA) advocate a dietary incorporation of 8 oz of seafood/week equating to approximately 250 mg of EPA and DHA per day [209]. Regarding alternative formulations and dosages that highlight the positive health benefits of omega-3 fatty acid supplementation, previous research indicated that an intake of 3.9 g/day over 16 weeks amplifies rates of mixed muscle, mitochondria, and sarcoplasmic protein synthesis in older adults pre- and postexercise [210]. Moreover, fish oil supplementation enriched with n-3 (approximately 2.2 g of EPA and 1.4 g of DHA) has demonstrated potential in mitigating the loss of body weight among advanced pancreatic cancer patients who have experienced severe weight loss [211]. Similarly, the administration of pure EPA (6 g per day) for 4 weeks was shown to contribute to weight stabilization over a 3-month period in pancreatic cancer patients [212]. Additionally, EPA supplements (3 g/day) have been associated with prolonged survival time in patients across various cancer conditions [213].

#### 9.2.2. Creatine

Creatine is a nitrogenous organic acid that is naturally present in common dietary sources, such as red meat, seafood, and poultry. The majority of creatine, approximately 95%, is stored within skeletal muscle, with the remaining 5% dispersed among the brain, liver, kidney, and the testes [214]. Intramuscular creatine serves a pivotal role in the phosphocreatine system. The benefits of creatine supplementation lie in its capacity to facilitate gain in muscle mass and thereby enhance muscular strength. This effect could potentially be attributed to its influence on high-energy phosphate metabolism, muscle protein kinetics, and growth factors [215,216]. According to the official stance of the International Society of Sports Nutrition (ISSN), creatine supplementation might contribute to minimizing the severity of injury, facilitating rehabilitation postinjury, and aiding athletes in enduring rigorous training regimens [217].

In the context of cancer cachexia, creatine supplementation has emerged as a plausible intervention. This is grounded in its potential to counteract the effects of impaired muscle protein synthesis and muscle degradation [215]. A study involving stage III or IV colorectal cancer patients demonstrated that those who received a creatine supplement regimen, consisting of 4 administrations of 5 g for the first week followed by 2 administrations of 2.5 g for 7 weeks, exhibited higher grip strength compared to a control cohort [218]. In a rodent model conducted by Wei and colleagues, creatine was found to shield against body weight loss and muscle wasting, leading to substantial improvements in grip strength among tumor-bearing mice [219]. Notably, the creatine treatment presented an ability to rectify mitochondrial dysfunction and morphological abnormalities, thereby safeguarding against cachectic muscle wasting, which could be achieved by inhibiting the aberrant overactivation of the ubiquitin proteasome system and autophagic lysosomal system [219].

#### 9.2.3. Branched-chain Amino Acids

It has been demonstrated that branched-chain amino acids (BCAAs), composed of leucine, isoleucine, and valine, have an ability to stimulate muscle protein synthesis while mitigating muscle wasting even in aging-induced muscle decline (i.e., sarcopenia) [220]. Circulating BCAAs can trigger protein synthesis, thus promoting muscle hypertrophy [213]. The enhancement of muscle protein synthesis can be attributed to the ability of BCAAs to upregulate the mTOR signaling pathway, which in turn fosters mitochondrial ATP production [221]. Additionally, the mTOR pathway collaborates with insulin and IGF-1 to increase intracellular BCAA uptake, promote protein synthesis, diminish protein degradation, and amplify cellular growth [222]. Furthermore, BCAA supplementation promotes the activation of PGC-1α activation and facilitates mitochondrial biogenesis and physiological function in cardiac and skeletal muscles through the mTOR pathway [223].

In contrast, BCAAs are normally oxidized as an energy source to generate ATP, which can potentially facilitate tumor growth under cancer-related catabolic environments [224]. Thus, it may be beneficial to administer BCAA supplementation to counteract the oxidation of BCAA observed in cancer cachexia. These beneficial outcomes linked to BCAA supplementation could be particularly relevant for patients undergoing cancer treatment or those in the precachexia phase. Previous studies demonstrated that patients with advanced intra-abdominal metastatic adenocarcinoma exhibited elevated whole-body protein synthesis and leucine balance after receiving a parenteral nutrition infusion of 50% BCAAs as compared with a formula of 19% BCAAs [225,226]. Additionally, a leucine-enriched diet amplified protein synthesis in skeletal muscle in walker 256 tumor-bearing mice through the activation of elF factors and/or the S6 kinase [227]. Another study utilizing a murine model to the explore impacts of leucine and valine discovered a significant reduction in body weight loss in tumor-bearing mice [228]. Although, the relationship between BCAA supplementation and cancer cachexia treatment is limited in terms of existing evidence, this avenue of research may warrant further exploration by virtue of its potential to enhance protein synthesis and combat the loss of muscle mass and muscle strength associated with cachexia. Further studies may be necessary to determine its efficacy when integrated with multiple treatments as part of a multimodal approach to cancer cachexia.

#### 9.2.4. Hydroxymethylbuterate

Another notable supplement that has demonstrated potential to ameliorate protein degradation under catabolic conditions is hydroxymethylbuterate (HMB), a bioactive metabolite formed from the decomposition of leucine, an essential BCAA [229]. The efficacy of HMB supplementation lies in its capacity to upregulate the mTOR signaling pathway in bolstering protein synthesis and concurrently dampen the proteasome signaling pathway, effectively counteracting muscle protein breakdown [230,231]. An illuminating study by Courel-Ibanez et al. revealed that HMB supplementation in sarcopenic individuals not only restrained muscle protein degradation but also augmented lean body mass and muscular power during a hospital-based rehabilitation and recovery program [232]. The prevailing recommendations for HMB dosage in sarcopenic individuals typically fall around 2–3 g/day (or 38 mg/kg/day), with no notable adverse effects [232,233]. It is worth noting that achieving the optimal dosage of HMB through a standard diet is challenging due to the limited presence of HMB in foods and the relatively low conversion rate of leucine to HMB [233,234].

In a study that examined the anticachectic effects of HMB, a combination of 3 g HMB, yielded a significantly higher lean mass in advanced cancer patients compared to a control cohort [235]. Notably, the anticachectic effect persisted for up to 24 weeks following the supplementation period. In a similar manner, HMB supplementation administered twice daily for 8 weeks produced positive outcomes in both body composition and clinical outcomes, including a lower incidence of overall health complications and reduced hospital readmission rates although statistical significance was not consistently achieved despite these favorable outcomes [236]. Additionally, HMB supplementation was found to lower serum CRP levels in malnourished elderly cancer patients [237]. Although the body of evidence regarding the efficacy of HMB supplementation in cancer cachexia remains relatively modest, the potential benefit it offers could complement the effects of other nutritional supplements in addressing muscle wasting. This synergy may hold promise for individuals undergoing cancer treatment.

### 9.3. Dietary Intake Interventions

#### 9.3.1. High-Fat Diets

High-fat diets, commonly referred to as ketogenic diets, are characterized by their extremely low carbohydrate content, moderate protein intake, and reliance on high-fat exogenous sources designed to increase blood free fatty acids and ketone bodies as alternative sources of energy to glucose. The primary proposed advantage of high-fat diets is in reducing the energy supply available to tumors while increasing the concentration of ketone bodies in the bloodstream. Tumor cells are unable to effectively utilize ketone bodies as a viable energy source [238,239,240,241] in contrast to healthy cells, which can utilize them to provide energy to skeletal muscle. A phenomenon known as “The Warburg Effect” involves the preference of tumor cells to predominantly rely on glucose for anaerobic energy production, reinforcing the adaptability of tumor cells in seeking alternative substrate sources [33].

By limiting the availability of glucose in an effort to starve tumors, the tumor cell growth might be constrained, potentially attenuating the progression of cancer cachexia and host catabolism [238,242,243]. A ketogenic diet implemented in C26 tumor-bearing mice resulted in preserved body, muscle, and carcass weight and markedly lowered tumor weight and plasma IL-6 levels, indicating a negative correlation between blood ketone bodies and tumor weight [242]. In addition, high-fat diets have been associated with increased survival time [244], reduced tumor burden with lower tumor growth [244,245], lowered tumor size [245], and a decrease in metastatic spread to various organs in tumor-bearing mice [245]. Among human cancer patients, high-fat diets produced similar outcomes, including a decrease in weight loss throughout cancer progression [243,246] and preserved muscle mass, ultimately improving quality of life [243]. These benefits of high-fat diets hold promise in regard to their integration into anticancer therapeutic strategies.

In cancer patients characterized with elevated systemic inflammation and impaired glucose oxidation and uptake, fat utilization may remain normal or even increase. This underscores the potential importance of maintaining a higher dietary fat-to-carbohydrate ratio in cancer patients [247,248,249]. However, it should be noted that a concern has been raised regarding the potential for this type of diet to trigger or worsen cachexia development due to the significant increases in plasma levels of total cholesterol and triglycerides [250,251,252,253]. Elevated plasma levels of these lipids are often detected in patients experiencing cancer cachexia as a result of heightened lipolysis [254]. A study by Clements et al. demonstrated metabolic improvements and reduced inflammation in tumor-bearing mice fed a high-fat diet, yet this diet also led to increased fat accumulation, exacerbated tumor progression, elevated metastasis, and reduced survival compared to a low-fat diet [253]. The potential benefits of high-fat diets might hinge on their application in cancer patients who are malnourished or struggle to maintain body weight. However, careful consideration must be given to avoid worsening the symptoms of cancer cachexia. Further research is necessary to establish the clinical efficacy of this dietary intervention as an anticancer cachexia strategy.

#### 9.3.2. Carbohydrate Diets

Research into the relationship between carbohydrate consumption and cancer progression has primarily revolved around manipulating dietary carbohydrate levels to potentially impede the proliferation of existing tumor cells. Since cancer cells have a greater reliance on substrate availability of glucose than do normal cells, investigations into a carbohydrate diet have predominantly concentrated on the impact of low-carbohydrate diets in ameliorating the tumor growth and progression of cancer cachexia. One study reported slower growth of carcinomas in murine and human models with a low-carbohydrate and high-protein diet compared to an isocaloric matched Western diet characterized by high-carbohydrate and low-protein content [255]. A subsequent study by the same group that employed an isocaloric diet comprised of low-carbohydrate (10%), high-protein (64%), and fat (26%) presented remarkable reductions in tumor growth while minimizing weight loss in tumor-bearing mice [255]. Encouragingly, this low-carbohydrate diet exhibited synergistic effects with established cancer therapeutic agents, such as Celebrex, in reducing tumor growth and incidence in a spontaneous mouse model of breast cancer [255]. These findings collectively underscore the safety and effectiveness of the low-carbohydrate diet in preserving body weight and curtailing tumor growth. Similar positive outcomes have emerged from studies that employed a diet with relatively low carbohydrate and moderately higher fat content. This diet has been linked to decreased tumor growth and attenuated cachexia in various experimental settings [238,242,256,257,258]. Additionally, while a ketogenic diet may not necessarily promote muscle mass hypertrophy, it could potentially counteract the loss of muscle mass by preserving existing muscle mass [259]. The trajectory of research regarding the incorporation of low-carbohydrate diets as part of an anticancer strategies should continue to explore the intricate balance among different macronutrients, with the goal of optimizing outcomes.

#### 9.3.3. Protein Diets

Adequate protein sources in one’s diet or through supplements are essential for maintaining positive protein balance, thus aiding in the enhancement of skeletal muscle mass. A potent catalyst for promoting muscular growth involves elevated plasma levels of amino acids derived from dietary protein sources. However, the information regarding protein intake in cancer cachexia is limited. A landmark study conducted by Muscaritoli et al. has laid the foundation for the practical clinical nutrition guidelines aimed at physicians, dieticians, nutritionists, and nurses caring for cancer patients. These guidelines advocate for a protein intake exceeding 1 g/kg/day and ideally reaching up to 1.5g/kg/day [260]. The rationale behind these protein recommendation stems from the fact that muscle protein synthesis remains responsive in cancer patients, and slightly higher provision of amino acids has demonstrated effectiveness [260]. Although the present clinical directives might suggest aligning protein regimens with the European Society for Clinical Nutrition and Metabolism (ESPEN) guidelines, as underscored by Muscaritoli and colleagues, additional evidence might be necessary to further support the significance of maintaining a positive protein balance in curtailing muscle mass loss in patients with cancer cachexia.

### 9.4. Pharmacological Interventions

#### Ghrelin Supplementation

**Figure 3:**
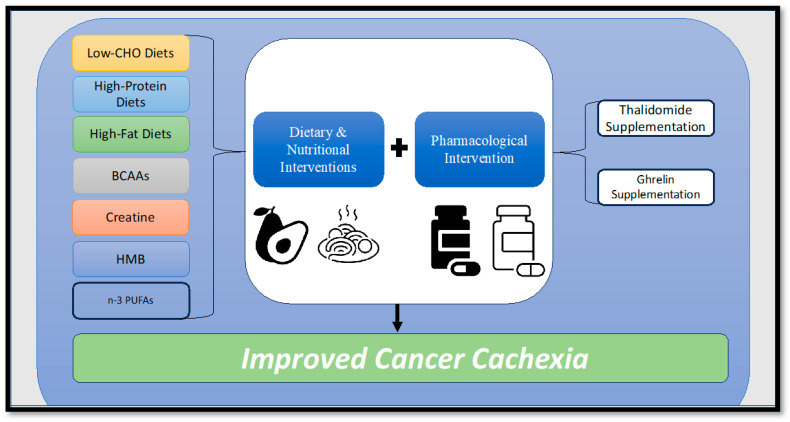
Diagram illustrating potential dietary, nutritional, and pharmacological interventions to combat cancer cachexia. BCAAs = Branched-chain amino acids, HMB = Hydroxymethylbutyrate, n-3 PUFAs = Omega-3 polyunsaturated fatty acids.

Anamorelin is another potential pharmacological option capable of enhancing appetite and mitigating the unfavorable outcomes of cancer cachexia [261]. Functioning as a ghrelin receptor agonist, anamorelin operates within the domain of ghrelin, a hormone responsible for influencing growth hormone secretion, appetite stimulation, and weight gain [262,263,264]. Notably, anamorelin’s oral formulation boasts a longer half-life of nearly 7–12 h compared to native ghrelin [265]. Although ghrelin participates in diverse biological processes, it is most recognized as the hunger hormone due to its role in sensing nutrients, triggering appetite, and initiating meals [266]. Research conducted by Pietra and colleagues unveiled compelling findings regarding anamorelin’s effect. Their investigation indicated that anamorelin administration at various dose levels (3, 10, or 30 mg/kg) led to significant increases in food intake and body weight in healthy rats compared to the control group [267]. Moreover, doses of 10 or 30 mg/kg correlated with notable elevations in growth hormone levels [267]. A follow-up study extended these observations, demonstrating heightened growth hormone levels in pigs following the oral administration of a single 3.5 mg/kg dose of anamorelin, along with increased IGF-1 levels following 7 days of daily anamorelin administration at 1 mg/kg/day [267]. In human studies, 12 weeks of anamorelin administration (100 mg) exhibited noteworthy outcomes. Specifically, this intervention led to a significant increase in lean body mass among patients grappling with late-stage non-small-cell lung cancer and cachexia as compared to the placebo group [268]. Further, a dosage of 100 mg of anamorelin administered for 24 weeks yielded improvements in body weight and alleviated anorexia symptoms in patients with non-small-cell lung cancer or gastrointestinal cancer associated with cancer cachexia [269]. Although anamorelin exhibits promise as a therapeutic agent in combating cancer cachexia, its application and efficacy in this context remain relatively limited. Further comprehensive research is indispensable to uncovering the full spectrum of its effects as an anticancer therapy. A depiction of these interventions was provided in Figure 3.

### 9.5. Gene Therapy

Gene therapy interventions have been collectively considered as a valuable approach in treating or preventing diseases by modifying genes to correct genetic defects. In the context of potential gene therapy for cancer cachexia, the aim is to modify the genes or genetic mutations within cancer cells to ameliorate their growth. One noteworthy study explored the use of recombinant adeno-associated viral vectors in a mouse model of cancer cachexia. The approach led to a reduction in Smad2/3 signaling downstream of ActRIIB and inhibited the expression of ubiquitin ligases MuRF1 and MAFbx [180]. Similarly encouraging results were observed from the introduction of a novel prototypic peptide called Pen-X-ACIP. The systemic delivery of Pen-X-ACIP into C26 mice resulted in a preservation of body weight and adipose tissue mass of the tumor-bearing mice [270]. Moreover, when Pen-X-ACIP was administered to human adipocytes, it decreased lipolysis, further supporting its potential as a gene therapy agent for cancer cachexia [270]. An innovative strategy for future gene therapy research could involve targeting the ectodysplasin A2 receptor (EDA2R). The activation of EDA2R signaling in tumor-bearing mice was found to promote skeletal muscle atrophy, while its deletion protected mice from muscle loss and functional decline [271]. Thus, targeting EDA2R may hold promise in the prevention of muscle loss in the progression of cancer cachexia. The potential gene therapy interventions in treating cancer cachexia discussed in this section may provide valuable insights for future research to delve deeper into promising approaches for addressing the issues of body weight and muscle mass loss associated with the progression of cancer cachexia.

### 9.6. Anti-Inflammatory Treatment

Within the realm of multimodal strategies for combating cancer cachexia, notable anti-inflammatory agents that target pro-inflammatory mediators hold promise in ameliorating this catabolic condition. These strategies work by inhibiting the production of pro-inflammatory cytokines, specifically TNF-α and IL-6 [272]. One such anti-inflammatory agent is the administration of thalidomide [273], which has demonstrated positive effects on body weight and appetite enhancement. In a previous study, the administration of 200 mg of thalidomide resulted in an average weight gain of 0.37 kg and an increase of 1.0 cm^3^ in arm muscle mass compared to the placebo group [274]. Similarly, oral administration of thalidomide (50 mg) in conjunction with megestrol acetate (160 mg) produced a significant improvement in body weight, quality of life, appetite, grip strength, and fatigue resistance, while substantially decreasing systemic TNF-α levels [272]. Notably, these improvements were more pronounced when both treatments were combined as compared to megestrol acetate administration alone [272,275]. Also, when anti-IL-6-antibody drugs were implemented in non-small cell lung cancer patients, there were significant improvements in anemia, cancer-associated cachexia, and fatigue resistance [276]. One such anti-IL-6 treatment, celecoxib, evaluated in clinical trials for cancer cachexia, produced remarkable improvements in lean body mass and grip strength [277]. While these anti-inflammatory agents targeting TNF-α and IL-6 have shown some positive results, further research is needed to fully understand their clinical efficacy in treating cancer cachexia.

### 9.7. Exercise Interventions

#### 9.7.1. Endurance Exercise Training

Exercise interventions have emerged as a pivotal strategy to counteract the detrimental impacts of cancer-induced muscle wasting. The empirical body of evidence on the efficacy of exercise interventions on cancer cachexia encompasses diverse approaches involving varying exercise types, intensities, and durations. A prevalent mode of low-intensity skeletal muscle engagement is aerobic exercise training, which has demonstrated a capacity to protect muscle mass in tumor-bearing mice [162,278,279,280,281,282]. Notably, in one study, even a solitary session of aerobic exercise through a treadmill exercise regimen triggered improved metabolic signaling, consequently contributing to improved muscle mass and physical functionality in tumor-bearing mice [283]. This principle extends to longer-term interventions, such as an 8-week treadmill running protocol conducted thrice weekly for 44 min at a range of 55–65% of VO2 max. This regimen was shown to result in noteworthy enhancements in VO2 max, maximal running speed, and a reduction in tumor cell proliferation and tumor growth in Walker 256 tumor-bearing mice compared to a sedentary control group [284].

Parallel findings underscore the ability of moderate-intensity treadmill running to counteract the deleterious effects of IL-6-induced mitochondrial remodeling and proteolysis even amidst high systemic IL-6 levels [285]. Translating these insights to human exercise protocols, the American College of Sports Medicine (ACSM) recommends that cancer patients should participate in at least 150 min of moderate or 75 min of vigorous exercise each week [286]. Although this recommendation serves as a foundational guideline for implementing aerobic exercise in cancer cachexia patients, it should be adapted to individual circumstances. However, the current exercise framework for cancer cachexia patients remains relatively constrained due to the scarcity of human randomized controlled trials and controlled trials specifically targeting cachectic symptoms [287]. This dearth of trials might be attributed to the relatively recent establishment of the international consensus framework for cancer cachexia [20] or the rapid progression of the condition that hinders well-designed studies. In exploring the link between inflammation and cachexia-induced muscle wasting, studies have shown that moderate-intensity endurance exercise can downregulate TNF-α, which plays pivotal roles in inflammation onset and regulation [288]. Similar results have been observed in cancerous mice, indicating the potential of aerobic exercise to decrease TNF-α expression in cachexic mice [289,290].

Research by Re Cecconi et al. explored the prospect that aerobic exercise prompts skeletal muscle to secrete myokines that could serve to combat cachexia. Their microarray analysis identified myokines including amphiregulin (AREG), natriuretic peptide precursor B (NppB), musclin, and fibroblast growth factor 18 (FGF18) as prominently induced by PGC-1α, which play a role in maintaining skeletal muscle and body fat [291]. Particularly, musclin demonstrated a significant correlation with PGC-1α expression in C26-bearing mice, suggesting its potential role in ameliorating muscle loss among patients unable to exercise [291]. In vivo research models have consistently demonstrated that aerobic exercise can help preserve muscle mass and function while reducing inflammation [291,292,293,294]. This was corroborated by a study that utilized aerobic training, which not only attenuated muscle wasting but also decreased tumor volume, mitigating levels of inflammatory markers such as the IL-10 and TNF-α ratio, along with IL-15 expression in skeletal muscle, in 4T1 cachectic breast cancer mice [197]. The culmination of these studies underscores the potential of aerobic exercise to counteract the progression of muscle wasting and weight loss in cachexia. Collectively, exercise interventions present a compelling avenue to address the challenges posed by cancer-induced muscle wasting, particularly through modes such as aerobic training, which hold promise in maintaining muscle mass, curbing inflammation, and fostering improved clinical outcomes in cancer cachexia.

#### 9.7.2. Resistance Exercise Training

Resistance exercise training has been established as a potent anabolic stimulus and could be considered a promising interventional strategy to combat cancer cachexia although the results from previous studies have been somewhat inconclusive. Investigations into resistance exercise protocols have utilized methods such as ladder climbs or electrical stimulation in rodent models [295,296] to replicate the effects of mechanical loading in humans. Notably, research by Testa and colleagues demonstrated that a ladder climbing protocol for resistance training led to decreased muscle atrophy in both the extensor digitorum longus and soleus muscles in tumor-bearing mice, which was attributed to the prevention of STAT3 phosphorylation, a reduction in IL-6, and decreased muscle lipid peroxidation mitigating loss of muscle strength, locomotion, and exploration capacity [297]. Similarly, electrical stimulation mimicking anabolic adaptations to resistance exercise in skeletal muscle was shown to attenuate cachexia-induced muscle wasting and protein depletion in tumor-bearing mice [298]. Despite these findings, the use of resistance exercise training as a countermeasure for cancer cachexia both in human and murine models remains limited. Alternative approaches, such as electric stimulation, have shown some promise in addressing muscle load and weakness associated with cachexia. Therefore, further research is needed to explore the potential of resistance exercise training, including methods such as weighted ladder climbs and electrical stimulation to mitigate the effects of muscle wasting associated with cancer cachexia.

During the progression of cancer cachexia, crucial signaling and metabolic pathways responsible for protein synthesis tend to be downregulated, leading to muscle loss [299]. Resistance exercise training can potentially counteract this decline by boosting protein synthesis and activating the mTORC1 pathway in patients with pancreatic cancer-induced cachexia [300]. Interestingly, when mTORC1 signaling is impaired due to cancer cachexia, a significant reduction (57%) in the protein synthesis rate occurs [301]. However, reactivation of the mTORC1 pathway in cachectic animals has been shown to partially reverse the loss of muscle mass and strength by 15–20% [301]. These findings underline the potential of resistance exercise training to counteract impaired protein synthesis and muscular strength in cancer cachexia patients.

#### 9.7.3. Concurrent Exercise Training

In terms of protein degradation in cancer cachexia, certain exercise programs have shown promise. For example, a treadmill-running protocol inhibited muscle mass loss in tumor-bearing mice through the suppression of the ubiquitin-proteasome pathway, enhancing hypoxia-inducible factor-1 alpha (HIF-1α) and phosphorylated 5′ adenosine monophosphate-activated protein kinase (AMPK), and prevented the deactivation of the mTOR pathway in the soleus muscle [302]. Comparable adaptations were noted in another study in which motorized wheel running led to the downregulation of distinct muscle proteolysis markers [F-box only protein 32 (Fbxo32), Trim63, MuRF1 and beclin-1] and partially restored autophagy with improved mitochondrial function, which in turn counteracted the decline in muscle mass and strength in C26 tumor-bearing mice [279].

Concurrent exercise training, which combines both aerobic and resistance training, is recommended as a safe and effective intervention to improve the quality of life for cancer patients throughout their treatment journey and even after cancer treatment [299,303,304,305]. Accumulating evidence suggests that the integration of both training modalities can yield notable benefits, fostering improvements in muscle mass and function. This may be achieved through mechanisms such as autophagy modulation and enhanced mitochondrial function, as evidenced in animal models [282]. Indeed, Wood et al., demonstrated that the combination of aerobic exercise and resistance exercise training can lead to significant reductions in systemic inflammation as indicated by lower spleen mass and plasma IL-6 levels in BALB/c mice [306]. In human cancer patients, a combination exercise protocol enhanced type II muscle fibers and the strength of the knee and elbow muscles while regulating the markers related to autophagy, the UPS system (FOXO3, MuRF1, Atrogin-1), and protein synthesis (mTOR, 3EBP1, S6rp) during chemotherapy and cancer cachexia [307]. It can be surmised that different exercise modalities seem to be effective in addressing protein degradation and the accelerated muscle wasting in cancer cachexia. In addition, Herrero and colleagues demonstrated that an 8-week program involving combined cardiorespiratory and resistance exercise training produced substantial enhancements in peak oxygen uptake (VO2 peak), strength, sit–stand test performance, and overall quality of life in breast cancer survivors [308]. Although the existing literature on the synergistic effects of concurrent aerobic exercise and resistance training remains limited, their potential is evident. Future research endeavors are necessary to delve deeper into this approach and establish its robustness in addressing key concerns associated with cancer cachexia.

## 10. Conclusions

Defining the profile of cachectic patients on both the molecular and clinical levels is an essential endeavor for delivering optimal and tailored treatment. Despite the elusive nature of the factors that impede progress in cancer cachexia improvement, adopting multifactorial treatment approaches holds the potential for significant clinical advancements in managing this metabolic syndrome among cancer patients. In the realm of clinical care, the effective implementation of a multimodal treatment hinges upon identifying the specific phase or stage in which a patient may be categorized during the cancer progression. This identification can serve as a catalyst for crafting a comprehensive treatment regimen encompassing exercise routines, nutritional support, and targeted administration of pharmacological agents. These interventions must be accompanied by clearly defined timepoints for periodic reassessment and evaluation, ensuring the continuous provision of cancer cachexia care. Integrating the aforementioned nutritional supplements and pharmacological agents with exercise interventions holds good prospects for countering catabolic processes by either preventing or ameliorating the actions of cachexia-inducing mediators. Future investigations are imperative for establishing the clinical efficacy of these proposed countermeasure tactics and engineering a readily applicable therapeutic intervention poised for seamless translation into clinical practice, thereby addressing the pressing unmet medical necessity in cancer cachexia treatment.
